# A Simulation Approach to Decision Making in IT Service Strategy

**DOI:** 10.1155/2014/829156

**Published:** 2014-03-26

**Authors:** Elena Orta, Mercedes Ruiz

**Affiliations:** Department of Computer Science and Engineering, University of Cadiz, C/Chile 1, 11003 Cadiz, Spain

## Abstract

We propose to use simulation modeling to support decision making in IT service strategy scope. Our main contribution is a simulation model that helps service providers analyze the consequences of changes in both the service capacity assigned to their customers and the tendency of service requests received on the fulfillment of a business rule associated with the strategic goal of customer satisfaction. This business rule is set in the SLAs that service provider and its customers agree to, which determine the maximum percentage of service requests that are permitted to be abandoned because they have exceeded the waiting time allowed. To illustrate the use and applications of the model, we include some of the experiments conducted and describe our conclusions.

## 1. Introduction

A convergence of a series of technologies and tendencies, such as high-speed Internet, web services, and service oriented architecture (SOA), has given rise to a new category of software developers with a radically different business model: web services providers. Web services are accessible via the Internet through open standards and service oriented architectures. Service providers design applications that can function as technology-independent reusable services, and the success of their businesses depends on services quality and satisfying customer expectations. Effective service management is itself a strategic asset of service providers, providing them with the ability to carry out their core business of providing services that deliver value to customers by facilitating the outcomes customers want to achieve [1].

There are different standards and reference process models that propose best practices for IT Services Management (ITSM) such as Information Technology Infrastructure Library (ITIL) [1], ISO/IEC 20000 International Standard [2, 3], and Capability Maturity Model Integration for Services (CMMI-SVC) [4], among others. ITIL is one of the process models used currently most often by organizations. Empirical works such as [5, 6] demonstrate the operational and strategic benefits obtained by companies that implement ITIL.

ITSM frameworks provide important benefits but their implementation in organizations is a complex process and managers have to make very important and difficult decisions. Due to the complexity and uncertainty in decision-making situations in this field, model-driven Decision Support Systems (*DSS*) help managers make better decisions. A model-driven DSS emphasizes access to and manipulation of quantitative models to support decision-making process [7]. Simulation models are an important type of quantitative model used in model-driven DSS. A simulation model can imitate the behavior of a system. It enables managers to modify model input parameters to examine the model outputs sensitivity and to realize analysis “*what if.*” It can capture underlying mechanism and dynamics of a system, which enables decision managers to effectively manage daily operations and make long term plans. It provides also a test-bed to asses changes in operations and managerial policies [8]. In [9], Power and Sharda present an overview of the research performed in the context of model-driven DSS. They show the applicability and utility of simulation-driven DSS to support decision making in different scopes.

The aim of this study is to analyze the use and applications of simulation modeling to help decision making in IT service strategy. We address the following two research questions.

- *Q1*: Is simulation modeling used to make decisions in IT service strategy?
- *Q2*: Is it adequate to use System Dynamics to solve IT service strategy problems?

The main contributions of this work that answer the research questions mentioned above are as follows:

- a study of the published papers that use simulation modeling to support decision making in IT service strategy scope. According to their application fields, the papers have been associated with the most suitable process of the ITIL Service Strategy module.
- System Dynamics that help evaluate the fulfillment of strategic goals for IT service providers and make decisions in the context of strategy management.

The rest of the paper is structured as follows. Next section presents general issues of simulation modeling and shows an overview of the works that apply simulation in the context of ITIL service strategy module.  Section 3 includes the description of the problem that our simulation model aims to resolve.  Section 4 describes our simulation model and explains some of the experiments performed to illustrate its use and applications. Finally, Section 5 summarizes this paper and presents our conclusions.

## 2. Simulation and IT Service Strategy

A simulation model is a mathematical model that represents a simplified form of a complex system whose equations are solved by simulation. The main goal of simulation models is to provide mechanisms for experimentation and behavior prediction, the resolution of questions such as “What would occur if …?” and learning more about the system represented, among others. They allow one to understand how systems behave over time and to compare their performance under different conditions. One of the main advantages of simulation models is that they allow one to experiment with different decisions and to analyze the results obtained in systems in which the cost, the time, or the risk of performing real experiments is high. Besides, simulation models permit the analysis of complex systems that are not possible to be represented with analytical models [10].

Simulation modeling is very frequently used to support decision making in different business areas, and it provides solutions to a wide range of issues at strategic, operational and tactical level [11–19].

There are different simulation approaches, such as, state-based process models, discrete-event simulation, System Dynamics, agent-based simulation, Petri-net models, queueing models, Monte Carlo simulation, probabilistic simulation, and traditional mathematical simulation [7]. The most appropriate approach depends on the nature of the problem to be solved. Specifically, System Dynamics mainly focus on strategic issues and policy analysis. This approach is considered appropriate when taking a distant perspective (meaning strategic) where events and decisions are seen in the form of patterns of behavior and systems structures [20].

This section provides an overview of the works that use simulation modeling to support decision making in the context of ITIL service strategy module. A search of various digital libraries and citation databases for papers that apply simulation modeling in this field has been performed. Our aim is not to offer an exhaustive study but to analyze if it is appropriate to apply simulation modeling in this scope.

The purpose of ITIL service strategy module is to design, develop, and implement service management as an organizational capability and as a strategic asset. This module processes are as follows: (a) strategy of IT services, (b) financial management for IT services, and (c) demand management. The objective of the strategy of IT services process is to ensure that service strategy is defined and maintained and achieves its purpose. It is responsible for defining strategic goals and the appropriate strategies for compliance. The purpose of the financial management for IT services is to secure the appropriate level of financing to design, develop, and deliver services that meet the strategy of the organization. Finally, the main aim of the demand management process is to understand, anticipate, and influence customer demand for services. This process works with the management capacity process to ensure that the service provider has enough capacity to meet this demand [21].

Below, we analyze the main application field of the papers referenced and associate them with the most suitable process of ITIL service strategy module.

### 2.1. Strategy for IT Services Process

Most works found in the scope of this process propose System Dynamics to help define the strategy of IT services. Gary et al. [22] present an overview of System Dynamics contributions in the strategy field focusing on why some firms are more profitable than others. The ideas introduced in this work can also be applied in the context of IT service strategy. The authors of [23, 24] focus on business objectives of organizations. Karapetrovic and Willborn [23] propose System Dynamics to describe links in a service organization and represent the interrelationships between business objectives, resources, and processes. On the other hand, Folgueras et al. propose to analyze different business objectives strategies and to determine the most adequate using Systems Dynamics [24]. Finally, the process modeling and simulation approach proposed in [25] help evaluate performance metrics, define an IT investment strategy, and select the optimal scenario for business and IT governance alignment.

### 2.2. Financial Management of IT Services Process

The following works apply simulation to analyze the cost of services. Gebauer [26] presents various System Dynamics models to study service behavior, resource allocation, customer perception, and reaction of competitors. The simulations results allow one to identify situations in which the cost of improving services exceeds the benefits. The framework for developing discrete-event simulation model proposed in [27] helps estimate serviceability, costs, revenue, profit, and services quality. Popkov and Karpov [28] introduce a simulation model that helps predict the costs of services, analyze the effects of business strategies, and define both IT infrastructure and services prices. The queuing model proposed by Villela et al. [29] helps evaluate the resource allocation that maximizes the benefits of service providers and minimizes the costs of services failures. The authors of [30] propose a probabilistic model that enables analyzing the expected change-related costs. In [31, 32] analytical models of service costs are presented. The authors of these works evaluate the effectiveness of their approach through simulation. In [31], Abrahao et al. introduce a cost model to analyze penalties due to violation and rewards received when the level targets are exceeded. In [32], the authors propose a model to analyze the costs of services considering different development teams and service providers. The model simulations help decide the optimal service provider.

Other more recent works study economic aspects of services in cloud computing environments [33]. In [34], an analytical model of hybrid cloud costs is presented. In this model the costs of computing and data communication are considered. The authors of [35] propose a mathematical model for supporting cloud services selection across multiple sources considering mainly cost and risk. Finally, in [36] Goiri et al. discuss an analytical economic model of resource provision in a federated cloud. The effectiveness of the models proposed in [34–36] is evaluated through simulation.

### 2.3. Demand Management Process

In [37] a service-demand-forecasting method that uses multiple data sources for improved accuracy is proposed. The authors present an advanced scenario simulation framework to analyze each customer service choice behavior and total service demand under an assumed condition. On the other hand, the authors of [38] focus on service multirequests from different users and propose a cooperative downloading strategy with multiclass request. The strategy provides different services according to users' expectations and helps service providers obtain the most benefit by making use of the repetition ratio of data and user-defined class. The simulation results indicate the benefits of the proposed scheme in terms of increasing service quality and benefit for service providers. Finally, Sen et al. [39] propose an analytical model for SLA formulation that is responsive to demand fluctuations and user preference variance, with the objective of maximizing organizational welfare of the participants. This formulation features a dynamic priority based price-penalty scheme targeted to individual users. Simulations performed using data from an existing SLA to provide evidence that the proposed dynamic pricing scheme is likely to be more effective than a fixed price approach are presented.

Our first research question is aimed at whether simulation modeling is used to make decisions in IT service strategy. The analysis of the papers above shows that these techniques are widely used in this context.

The second research question is aimed at whether it is adequate to use System Dynamics to solve IT service strategy problems. The authors of [20] emphasize that System Dynamics focus mainly on strategic issues and policy analysis. Besides, although the referenced papers show that different simulation approaches have been used in this field, most of papers found in the context of strategy for IT service process propose System Dynamics to define service strategy, strategic goals, and analysis policies [22–24]. Instead, in the context of the financial management of IT services and demand management processes, other simulation approaches such as analytical models or discrete-event simulation are more used.

In the following sections, we present a System Dynamics model built to help service providers in the decision-making process to define their strategic goals.

## 3. Problem Description

One of the main objectives of the ITIL strategy of IT services process is to define the strategic goals of organizations and the appropriate strategies for compliance. Strategic goals can be expressed in the form of different managerial business rules that aggregate different business rules that affect the structure and behavior of the services [40]. Thus, the fulfillment of the business rules determines the fulfillment of the strategic goals. In this paper we focus on the business rules that indicate restrictions about services behavior. These business rules figure in the contracts service providers and their clients sign called service level agreement (SLA) [41].

For the purpose of this study, we have considered a hypothetical e-commerce company that sells products on the Internet. The e-commerce company is a distribution company that buys the products to their manufacturers and sells them to their customer through the company website.

In SOA approach, the tasks that validate credit card details and verify that company's customers possess enough credit to make the purchase are handled by the credit card validation service provided by a banking validation service provider. In this context, the e-commerce company and the service provider sign a SLA in which issues such as service capacity contracted, service availability, service response time, service abandon rate, key performance indicators, and operation or billing model are set, among others. The billing model and the quantity the company has to pay to the service provider depend on the values estimated and specified for the SLA parameters. Errors in the estimation of these parameters will have a direct effect not only on the bill but also on the fulfillment of both business rules and strategic goals of the service provider.

With the aim of helping service providers in the decision-making process to define their strategic goals, a simulation model is built to allow evaluating the effects of different service capacities and service request tendencies on the fulfillment of our business rule and hence the strategic goal customer satisfaction.

In this study, we have focused mainly on the business rule that specifies the maximum percentage of service requests that are permitted to be abandoned (15%) because they have exceeded the waiting time established. Consequently, we have considered the SLA parameters most frequently used to define this business rule [1].

- *Service Capacity Contracted*. Maximum capacity of the credit card validation service contracted by the company.
- *Service Response Time*. Maximum response time of the credit card validation service. If this time is exceeded, the service request is rejected.
- *Request Rejection Rate (Our Business Rule)*. Maximum percentage of service requests that are permitted to be rejected because they have exceeded the maximum service response time established.

It is important to notice that we have focused only on the service requests rejections due to the delay in the response of the service for more than the maximum response time allowed by the company. We have not considered other reasons for service requests rejections such as server down, incorrect credit card data, and insufficient balance of the credit card, among others.

## 4. Simulation Model

This section describes the simulation model built to analyze the problem described in the above section. For this, we have followed Kellner et al.'s proposal to describe a simulation model [10] and Martínez and Richardson's methodology for simulation model building [42]. The main components of a simulation model are the scope and purpose of the model, the abstraction of the system, the input parameters, and the output variables [10].

The simulation approach chosen for model building is System Dynamics and the simulation tool used is AnyLogic [43], a multiparadigm simulation environment.

### 4.1. Purpose and Scope of the Model

The scope of the model is ITIL strategy of IT services process. The model purpose is to help service providers evaluate the fulfillment of the business rule that specifies the maximum rejection percentage for service requests allowed.

### 4.2. Input Parameters

Input parameters allow the configuration of different simulation scenarios to analyze the fulfillment of the business rule contracting different service capacities and considering various tendencies of service requests. The input parameters included in this study are classified as follows.

(a) Parameters modeling the tendency of the service requests that the service provider receives.

- *Requests Received Rate.* It represents the rate of service requests received that depends on the tendency of the orders made by the company's customer through the website. In the case of study, we have considered the patterns of behavior of customer's orders after the lunching of a special offer shown in Table 1.

*Requests Received Rate*,* Initial Value, Pulse Height, Pulse Time, Pulse Width, Ramp Slope, Ramp Time, and Ramp Length* are input parameters of the model that allow defining the patterns of behavior of customer's orders considered. It is possible to include in the simulation model other functions to represent other different tendencies of the customer's orders.

(b) SLA parameters service provider and e-commerce company sign (see Section 3).

- *Service Capacity Contracted.* It represents the service capacity contracted by the customer company.
- *Service Response Time.* It represents the maximum response time of the service.
- *Requests Rejection Rate.* It represents the maximum percentage of service requests received that are permitted to be rejected.

(c) Credit card validation service management parameters.

- *Service Capacity.* It represents the service capacity that service provider assigns to the customer company.

### 4.3. Output Variables

The main output variables that provide information about the purpose of the model and are helpful to understand the behavior of system are as follows:

- *Business Rule Fulfillment. *It represents the deviation between the service requests that have been rejected by the system and the maximum rejection rate allowed by the business rule (value of the SLA parameter* Requests Rejection Rate*).
- *Requests Received. *It represents the number of service requests that the service provider receives. The value of this variable depends on the value of the input parameter* Requests Received Rate. *
- *Requests Validated. *It represents the number of service requests received that have got an answer without exceeding the maximum response time established.
- *Requests Rejected. *It represents the number of service requests received that have been rejected because they exceeded the maximum response time established.

### 4.4. Model Conceptualization

**Figure 1:**
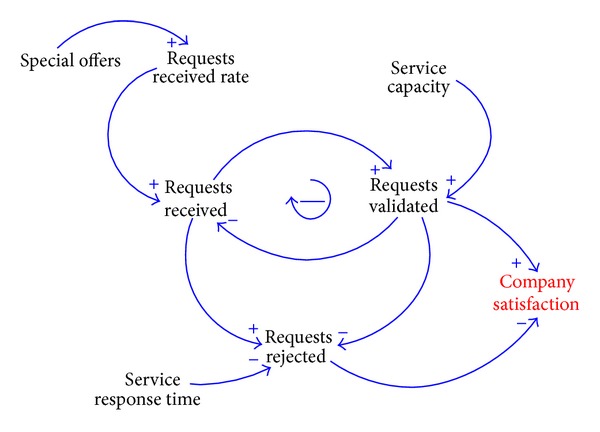
Causal loop diagram.

In order to represent the main elements of the system modeled and their cause and effect relationships, we have created the causal loop diagram presented in Figure 1.

The cause and effect relationships displayed in Figure 1 are as follows: (a) the number of* Requests Received* depends on the* Requests Received Rate* that represents the tendency of the company customer's orders received; (b) the number of* Requests Validated* depends on the number of* Requests Received* and the service capacity that service provider assigns to the customer company; (c) the number of* Requests Rejected* depends on the number of* Requests Received*, the* Service Response Time* (maximum service response time allowed), and the number of* Requests Validated*; (d) the strategic goal customer satisfaction depends on the number of* Requests Validated* and the number of* Requests Rejected*. The input parameters of the model are the activity elements upon which the fulfillment of the business rule can be assured.

Once the model has been conceptualized, it has to be formalized as a mathematical model. For this, we have used stock and flow diagrams, also known as Forrester diagrams [44]. These diagrams help us represent the model structure, assign the equations to this structure, and identify the relationships between the variables involved. They represent the most important system variables, that is, those ones whose behavior we wish to observe, as stock variables. The variables that represent how stock variables change through time are known as flow variables. Auxiliary variables are the rest of elements in the process that have influence upon it.

In the model proposed, the output variables* Requests Received*,* Requests Validated*, and* Requests Rejected* have been modeled as stock variables whose behavior is controlled by the flow variables* Requests Received Rate*,* Validation Rate*, and* Rejection Rate*.

### 4.5. Model Experimentation

To illustrate the use and applications of the model, in this section we describe and analyze some of the experiments conducted. These experiments are classified as follows.

- Experiments that allow analyzing the fulfillment of the business rule and the service behavior by varying both the service capacity that service providers allocate to the company and the tendency of customer's orders that determines the tendency of service requests that service provider receives.
- *Optimization Experiments*. These experiments combining simulation model with the AnyLogic optimization tool to find the best values of decision variables (input parameters of the simulation model) which yield the optimal service performance. The AnyLogic optimization tool is based on the* OptQuest* optimization engine which uses metaheuristic optimization algorithms, mainly scatter search [43].

#### 4.5.1. Experiments to Analyze the Fulfillment of Business Rule and Service Behavior

**Experiment 1**

The aim of this experiment is to analyze the fulfillment of the business rule and the system behavior with an initial system configuration determined by the input parameters values indicated in Table 2.

**Figure 3:**
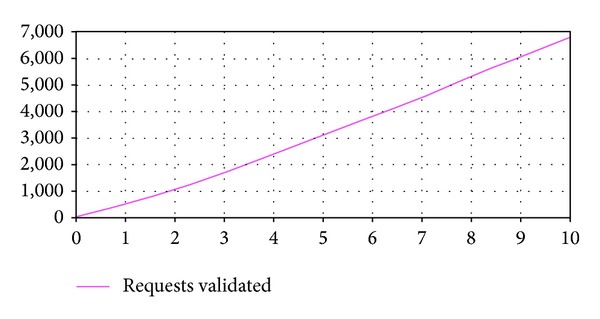
Output variable* Requests Validated* (E1).

The simulation results indicate that, with the input parameters configuration considered in this experiment, the service provider compliances the business rule established in the SLA. They also indicate that no service requests are rejected during all the simulation period because of exceeding the maximum response time established. On the other hand, Figure 3 shows the service requests validated within the expected response time. It can be observed a gradual increment of this output variable value and that at the end of the simulation period it reaches a value of 6.798 requests.

**Experiment 2**

The aim of this experiment is to determine the lowest service capacity that guarantees the fulfillment of the business rule. For this, several model simulations were run setting the input parameter* Service Capacity* to a lower value than the service capacity contracted by the company (900 requests/minute). The simulation results indicated that the lowest service capacity is 739 requests/minute. With this service capacity, 6.792 service requests are validated and 6 service requests are rejected at the end of the simulation period.

In this case, the service provider can assign less service capacity than the one contracted by the company, with the certainty that the business rule is met. Therefore the unnecessary service capacity can be used to satisfy the service requests of other customers.

**Experiment 3**

**Figure 4:**
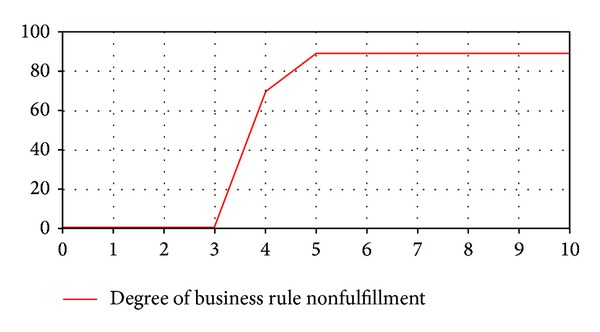
Output variable* Business Rule Fulfillment* (E3).

**Figure 5:**
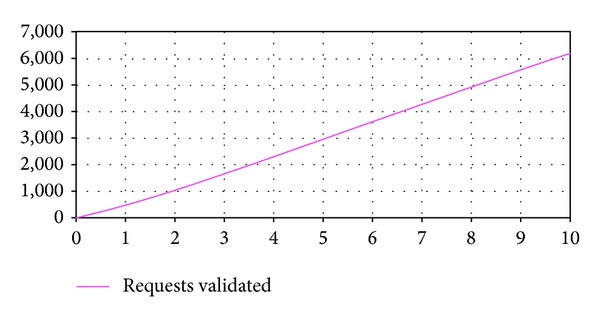
Output variable* Requests Validated* (E3).

**Figure 6:**
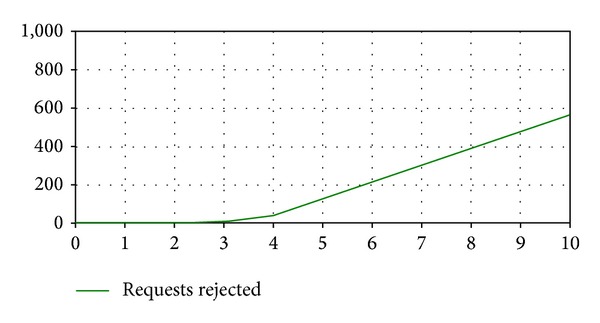
Output variable* Requests Rejected* (E3).

The main objective of this experiment is to analyze the service performance with service capacities that do not meet the business rule. Figures 4, 5, and 6 represent the values of the output variables obtained with a service capacity equal to 650 requests/minute.  Figure 4 shows the behavior of output variable* Business Rule Fulfillment* with this capacity. It is observed that initially the business rule is met, but from a specific point in time to the end of simulation this rule is not satisfied anymore. Besides, the degree of the business rule nonfulfillment varies during the simulation period, and from a particular point in time (5 minutes) to the end of the simulation, this variable reaches its highest value that it is equal to 89 requests.

Figures 5 and 6 display the output variables* Requests Validated* and* Requests Rejected*,respectively.  Figure 5 shows that the variable* Requests Validated* experiments a gradual increment and at the end of the simulation its value is 6.209 requests.  Figure 6 indicates that service requests begin to be rejected at a specific point in time (3 minutes), and 567 service requests have been rejected at the end of the simulation.

The analysis of the results of this experiment indicates that to fulfill the business rule it would be necessary to modify the service capacity assigned to the company over time. Initially, a service capacity equal to 650 requests/minute is adequate, but in different time periods higher service capacities are needed to ensure the fulfillment of the business rule. Thus, model simulations provide information that help service provider decide how to manage service capacity to meet the business rule and to optimize his resources.

**Experiment 4**

**Figure 7:**
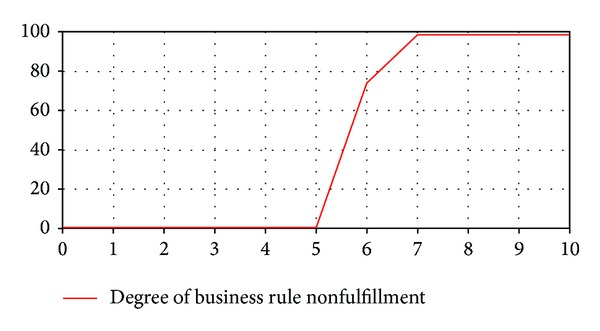
Output variable* Business Rule Fulfillment* (E4).

The purpose of this experiment is to evaluate the service behavior with the system configuration considered in Experiment 1, but varying the characteristics of the ramp that models the tendency of service requests as follows: (a)* RampTime* = 1 minute, (b)* RampLenght* = 6 minutes, and (c)* RampSlope* = 100. The degree of business rule nonfulfillment in this scenario is displayed in Figure 7. It is observed that the business rule is not satisfied from a specific point in time (5 minutes), and the degree of nonfulfillment varies over time.

Comparing the results of this experiment with Experiment 3, it is observed that, with a higher service capacity and increasing ramp slope, the nonfulfillment of the business rule starts later but it reaches a higher degree.

The results of the experiments above described show that the compliance of the business rule depends on both the service capacity assigned to the company and the tendency of the company's service requests. Thus, if the service provider could estimate the future demand of the customer company's service requests, they would know what service capacity to assign it to guarantee the compliance of the business rule.

#### 4.5.2. Optimization Experiments

The optimization experiments have been performed with the system configuration considered in Experiment 1, varying the values of the input parameters* Service Capacity* (between 500 and 900) and* Requests Rejection Rate* (between 0.10 and 0.20).  Table 3 summarizes the results of these experiments. It shows the service capacity that the service provider would have to assign to the customer company to meet the optimization objectives established. Besides, it shows the business rules that the service provider would satisfy with those capacities.

## 5. Conclusions

The first research question asked whether simulation modeling is used to support decision making in the IT service strategy context. This paper explores the use of simulation modeling in this scope and the works founded show that different simulation approaches have been used. According to their application fields, these works have been associated with the most suitable process of ITIL Service Strategy module.

The second research question asked whether it is adequate to use System Dynamics to solve IT service strategy problems. The main contribution of this work is a System Dynamics model in the scope of ITIL strategy of IT services process that helps service providers in the decision-making process to define their strategic goals. The simulation model allows analyzing the outcomes of changes in the service capacity, which a service provider assigns to a specific customer, and in the tendency of customer's service requests on the fulfillment of one of the business rules associated with the strategic goal customer satisfaction. This business rule is reflected in the SLA that the service provider and the customer sign and determines the maximum percentage of service requests that are permitted to be rejected because they have exceeded the response time established.

To do that, different simulation scenarios were configured by varying the service capacity and the tendency of service requests received. The main objectives of the experiments described in this study are as follows: (a) to evaluate the fulfillment of the business rule with the service capacity contracted by the customer, (b) to determine the lowest service capacity that ensures the fulfillment of the business rule, and (c) to analyze the service behavior by varying the service capacity assigned to the customer and the tendency of service requests received. The dynamic feature of the simulation model helps analyze the fulfillment of the business rule and the service behavior through time and determine the adequate moment to change the service capacity to guarantee the business rule fulfillment.

On the other hand, several optimization experiments were performed that allow determining the best values of decision variables that meet the following optimization objectives: (a) to minimize the service requests rejected, (b) to maximize the service requests validated, and (c) to minimize the nonfulfillment of the business rule.

The main objectives of our further works are as follows:

- extend the functionality of the simulation model presented in this work to solve more complex problems in the context of the IT service strategy.
- develop simulation models to help in decision making in different domains of IT service management processes. For this, different simulation approaches can be applied;
- apply the simulation models built in real companies to help calibrate and validate them. The usage of these models will provide important benefits for the companies.

## Figures and Tables

**Figure 2:**
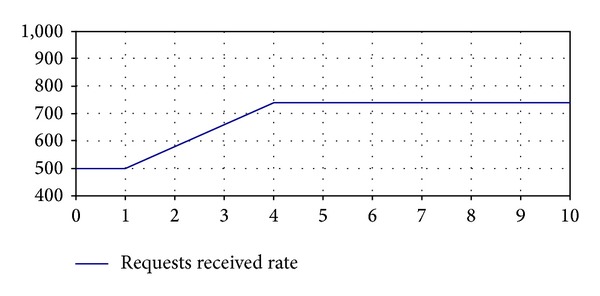
Pattern of behavior for the input parameter* Requests Received Rate* (E1).

**Table 1:** Patterns of behavior of customer's orders.

Orders tendency	Description
Const	After the launching of a special offer, the number of orders received experiments a rapid growth and it remains constant as its peak value. *Requests Received Rate* = *Initial Value* + *PulseHeight* ∗ Pulse (*PulseTime*, *Sim Period*)

Ramp	The increment of the number of orders is not step-shaped but gradual; hence, it has been modeled as ramp. *Requests Received Rate* = *Initial Value* + Ramp (*Ramp Slope*, *Ramp Time, Ramp Length*)

Pulse	The growth that the number of orders stays as its peak value for a certain amount of time and then descends gradually. *Requests Received Rate* = *Initial Value* + *PulseHeight* ∗ Pulse (*PulseTime*, *PulseWidth*)

**Table 2:** Configuration of the input parameters of the simulation model (E1).

Input parameters configuration	
*Request Received Rate*.* *The pattern of behavior of orders received after the launching of a special offer is modeling by the ramp shown in Figure 2. The following input parameters configure this ramp:	
(i) *Initial Value: *500 orders/minute.	
(ii) *Ramp Time: *1 minute.	
(iii) *Ramp Length: *4 minutes.	
(iv) *Ramp Slope: *80.	
SLA parameters:	
(i) *Service Capacity Contracted: *900 requests/minute.	
(ii) *Service Response Time: *15 seconds.	
(iii) *Requests Rejection Rate:* 15%.	
*Service management parameters: *	
*Service Capacity: Service Capacity Contracted *(900 requests/minute).	

**Table 3:** Optimization experiments.

Exp	Optimization objective	Service capacity	Requests rejection rate
1	To assure the fulfillment of the business rule and to minimize the service requests rejected	800	0.175

2	To assure the fulfillment of the business rule and to maximize the service requests validated	854.5	0.102

3	To minimize the nonfulfillment of the business rule	739	0.136
